# The Effect of Remimazolam on the Baseline TOF Ratio: A Prospective, Clinical Study

**DOI:** 10.1155/anrp/9990140

**Published:** 2024-11-28

**Authors:** Tomoko Yuruki, Masafumi Fujimoto, Naoyuki Hirata

**Affiliations:** Department of Anesthesiology, Graduate School of Medical Sciences, Kumamoto University Hospital, Kumamoto University, 1-1-1, Honjyo, Cyuoku, Kumamoto-City 860-8556, Kumamoto, Japan

## Abstract

**Background:** Remimazolam is a newly developed benzodiazepine. Early recovery from anesthesia because of its ultra-short acting effect and less hemodynamic side effects has been reported as the specific advantages of remimazolam. Therefore, the maintenance of anesthesia with propofol may be sometimes switched to remimazolam anesthesia maintenance during surgery because of the risk of delayed awakening and unstable hemodynamics. In the present study, to determine the influence of switching anesthesia from propofol to remimazolam on the baseline TOF ratio, the TOF ratio under remimazolam anesthesia maintenance without any neuromuscular blocking agents was compared to that calibrated after induction of general anesthesia with propofol.

**Methods:** Twelve patients scheduled for elective surgery under general anesthesia in the supine position were investigated. After induction of general anesthesia with remifentanil and propofol, a supraglottic airway was inserted without neuromuscular blockade, and TOF stimulation every 15 s at the adductor pollicis muscle was started with acceleromyography. After stable baseline responses to TOF stimulation being obtained for at least 10 min under propofol anesthesia, the anesthetic agent was switched to remimazolam and TOF stimulation every 15 s was maintained for a further 60 min without any interruption. In each case, the averaged TOF ratio during the last 10 min of TOF monitoring was compared to that during the 10 min immediately before the beginning of remimazolam infusion using a paired *t*-test.

**Results:** There were no significant differences in the TOF ratios before and after switching anesthesia to remimazolam (1.07 ± 0.03 vs. 1.07 ± 0.03, *p*=0.325).

**Conclusion:** Switching anesthesia from propofol to remimazolam does not affect the baseline TOF ratio.

## 1. Introduction

Recently, it was not demonstrated that adherence to the recommended train-of-four (TOF) ratio of 0.9 before extubation was associated with better pulmonary outcomes [1]. The overestimation of recovery from neuromuscular blockade of acceleromyography, the most frequently used neuromuscular monitoring device, due to its property of indicating the TOF ratio > 1.0 is considered as one of the possible explanations for this finding [1, 2]. Therefore, anesthesiologists are recommended to note the baseline TOF ratio before administration of neuromuscular blocking agents at the induction of general anesthesia and confirm recovery of the TOF ratio to at least 90% of the baseline value before extubation [3].

Remimazolam is a newly developed benzodiazepine. Early recovery from anesthesia because of its ultra-short acting effect and less hemodynamic side effects has been reported as the specific advantages of remimazolam [4, 5]. Therefore, the maintenance of anesthesia with propofol, the most common intravenous anesthesia, may be sometimes switched to remimazolam anesthesia maintenance during surgery because of the risk of delayed awakening and unstable hemodynamics. On the other hand, the detailed effects of remimazolam on neuromuscular function have not been reported. Thus, the influence of switching anesthesia to remimazolam on the baseline TOF ratio remains unclear.

The purpose of this study is to determine the influence of switching anesthesia from propofol to remimazolam on the baseline TOF ratio. We hypothesized that switching anesthesia to remimazolam does not affect the baseline TOF ratio. To test this hypothesis, the changes in the TOF ratio were compared between remimazolam anesthesia maintenance and propofol anesthesia maintenance without any neuromuscular blocking agents. To eliminate the risk of error due to TOF monitoring variability between patients, changes of the TOF ratio due to the two anesthetic agents were observed within the same patient in the present study.

## 2. Methods

### 2.1. Ethical Considerations

This prospective clinical trial was conducted according to the Declaration of Helsinki, with the approval of the ethics committee of Kumamoto University Hospital (protocol Rinri-2749; July 6, 2023). All participants gave their written informed consent before starting the trial. The trial was registered in Japan Registry of Clinical trials (jRCT1071230039, July 10, 2023; principal investigator: Masafumi Fujimoto, https://jrct.niph.go.jp/re/reports/detail/68710) before the first patient was enrolled.

### 2.2. Inclusion and Exclusion Criteria

The following patients were assessed for eligibility to participate: age over 18 years, scheduled for elective surgery under general anesthesia in the supine position, except for head, neck, upper limb, and abdominal surgery. All patients were included in the American Society of Anesthesiologists' physical status Class I or II. Exclusion criteria included known or suspected difficult airway including obesity (body mass index [BMI] ≥ 30.0 kg/m^2^); history of gastroesophageal reflux or high risk for aspiration; contraindications to the use of benzodiazepines; neuromuscular disorders.

### 2.3. Anesthesia and Neuromuscular Monitoring

We followed the methods of Fujimoto et al. [6]. Premedication was not used in any patients. Upon entry into the operating theater, routine monitoring was applied to all patients, including electrocardiography, pulse oximetry, noninvasive blood pressure and end-tidal CO_2_ measurement, and anesthetic depth monitoring (BISx module NK, Nihon Kohden, Tokyo, Japan), and an intravenous cannula was inserted into a forearm vein for the administration of routine anesthetics and study drugs. A continuous infusion of remifentanil and propofol was used to induce and maintain general anesthesia. A target-controlled infusion technique (TCI pump; TERUMO, Tokyo, Japan) was used to adjust the propofol infusion rate, using a target concentration of 2.0–5.0 *μ*g/mL to maintain the BIS value in the range of 40 to 60. Remifentanil was continuously infused and adjusted to 0.1–0.5 *μ*g/kg/min. After the eyelash reflex was lost, the patient was ventilated with a mask for a few minutes, a supraglottic airway (i-gel, Intersurgical, Wokingham, Berkshire, United Kingdom) was inserted without neuromuscular blockade, and mechanical ventilation was started. The anesthetic agents and doses were adjusted as necessary by the attending anesthesiologists to provide optimal patient care, with blood pressure management within 20% of baseline values. A bolus injection of fentanyl 1.5–2.0 *μ*g/kg during periods when neuromuscular data were not being collected was also permitted.

**Figure 1:**
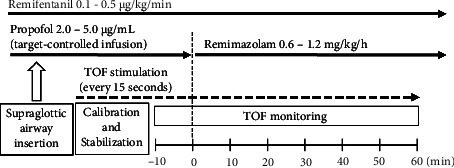
Schematic diagram of the induction of general anesthesia and data collection. TOF, train-of-four.

After anesthesia was induced, a TOF-Watch SX (Nihon Kohden, Tokyo, Japan) was placed on the arm opposite to that with the blood pressure cuff for neuromuscular monitoring at the adductor pollicis muscle. The neuromuscular data of the adductor pollicis muscle were collected by a transducer that was attached to the thumb; the data were then transferred in real time to a computer and recorded using the TOF-Watch SX monitoring program. Neuromuscular monitoring was performed according to the guidelines of Good Clinical Research Practice in pharmacodynamic studies of neuromuscular blocking agents [7]. With the patient's study arm immobilized, a preload was applied to the thumb with a hand adapter. TOF stimulation of the ulnar nerve was applied through surface electrodes at the wrist every 15 s for at least 10 min without preceding tetanic stimulation. When a stable response to TOF stimulation was achieved (variation less than 5%), the built-in calibration function (CAL 2) ensured calibration and supramaximal stimulation. Once stable baseline responses to TOF stimulation were obtained for at least 10 min, TOF stimulation was continued every 15 s for a further 10 min. The propofol infusion was then discontinued, with anesthesia maintained by continuous remimazolam infusion, and TOF stimulation every 15 s was continued without interruption for 60 min (Figure 1). The remimazolam infusion rate was adjusted at 0.6–1.2 mg/kg/h to maintain the BIS value in the range of 40–60.

While neuromuscular monitoring was being performed, nasal temperature, reflecting central body temperature, was measured and maintained at ≥ 35°C. Peripheral body temperature was also measured continuously by a thermistor at the thenar eminence, maintaining it ≥ 32°C with a forced air-warming device.

### 2.4. Statistical Analysis

Considering the measurement variations of TOF monitoring, for each case, the TOF ratios were measured at the shortest interval of 15 s and averaged every 10 min, and then the averaged TOF ratio during the last 10 min of the TOF monitoring and that during the 10 min immediately before the remimazolam administration were compared using the paired *t*-test. The first twitch responses to the TOF stimulation were similarly compared between before and after remimazolam administration.

The recovery of the TOF ratio recommended to extubate patients is commonly more than 90% of the baseline value [3, 8, 9]. In the present study, therefore, a clinically relevant change of the TOF ratio was defined as a 10% decrement. As the average baseline TOF ratio and standard deviation (SD) evaluated by acceleromyography have been reported to be 1.10 ± 0.09 [3], the sample size needed to detect the decrement of 0.11 in the TOF ratio with an SD of 0.09 was calculated. A prior power analysis showed that, with *α* = 0.05 and *β* = 0.20, the sample size required in the present study was eight. Given that calculation, it was decided to recruit 15 patients, taking into account missing data.

Data are expressed as mean ± SD values. The R statistical package version 3.4.1 (R Foundation for Statistical Computing, Vienna, Austria) was used for all statistical analyses, including the sample size calculation, and significance was defined as a *p* value < 0.05.

## 3. Results

**Figure 2:**
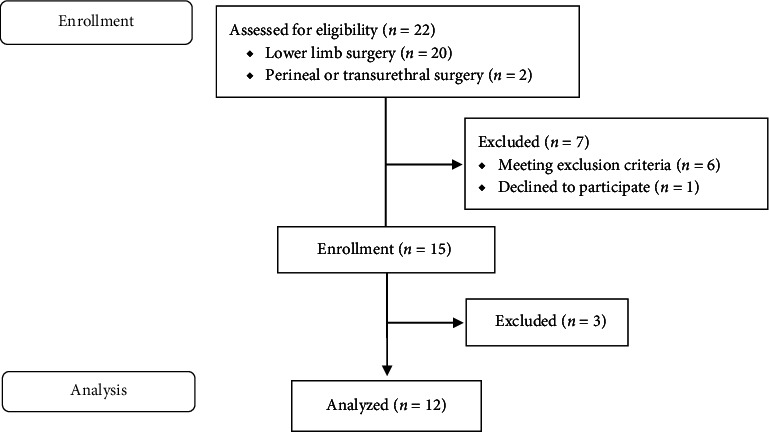
Flow diagram of study participation.

The study was performed at Kumamoto University Hospital, with patients enrolled from July 24, 2023, to November 1, 2023. Of the 22 patients assessed for study eligibility, seven were excluded. Two of the remaining 15 patients were excluded because rocuronium administration was needed. One was for movement during induction of general anesthesia, and the other was for tracheal intubation because of a change in the airway management plan. In addition, another patient was excluded because of technical difficulties in the recording device for neuromuscular monitoring. Therefore, a total of 12 patients were included in the final analysis (Figure 2). The patients' basic demographic data are shown in Table 1. There were no patients with diseases interfering with the pharmacodynamics of study drugs, such as cirrhosis, hepatitis, cholestasis, heart failure, and renal dysfunction. In all cases, surgery was started before switching anesthesia to remimazolam and some patients received a bolus injection of fentanyl prior to starting surgery. There were no patients required administration of anesthetics deviated from the protocol.

**Figure 3:**
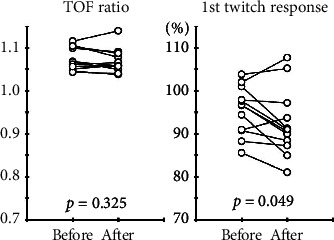
Changes in the TOF ratios and the first twitch responses between before and after remimazolam administration. There are no significant differences in the TOF ratios between before and after remimazolam administration. In contrast, a significant decrement in the first twitch response is seen. TOF, train-of-four.

**Figure 4:**
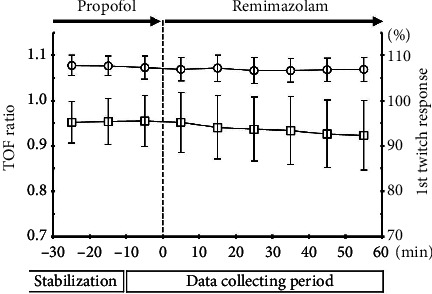
The averaged TOF ratios and first twitch responses every 10 min during data collection. The circles represent the TOF ratios, and the squares represent the first twitch response. The whiskers indicate standard deviations.

There were no significant differences in the TOF ratios between before and after remimazolam administration (1.07 ± 0.03 vs. 1.07 ± 0.03, *p*=0.325) (Figure 3). In contrast, there was a significant decrement in the first twitch response (95.5 ± 5.7% vs. 92.4 ± 7.8%, *p*=0.049). The averaged TOF ratios and first twitch responses every 10 min during data collection are shown in Figure 4.

There were no complications or adverse events related to the study during the surgery or in the postoperative period including muscle pain in the fingers or hands on the monitoring side.

## 4. Discussion

To the best of our knowledge, the present study is the first to investigate the influence of switching anesthesia from propofol to remimazolam on the baseline TOF ratio. In the present study, no significant changes between the TOF ratio under remimazolam anesthesia maintenance and that calibrated after induction of general anesthesia with propofol were demonstrated, suggesting that the baseline TOF ratio was consistently available even after switching anesthesia from propofol to remimazolam. Some benzodiazepines, such as diazepam and midazolam, are known to have centrally acting muscle relaxant effects induced by reduction of the polysynaptic reflex in the spine, which is thought to relate to the potentiation of some gamma-aminobutyric acid (GABA)–ergic inhibitory processes [10]. The effect of benzodiazepines on the TOF ratio has not been reported, but there are a few reports investigating their interaction with neuromuscular blocking agents [11, 12]. According to Olkkola and Tammisto, the infusion rate of rocuronium necessary to produce a constant 90% block in the first twitch response to TOF stimulation from the control value did not differ significantly between patients receiving propofol and those receiving midazolam [11]. Driessen et al. reported significantly delayed recovery of the twitch response after administration of vecuronium and artacurium in patients receiving midazolam compared with those receiving diazepam [12]. Considering these reports, the interaction of each benzodiazepine including remimazolam with neuromuscular blocking agents and their effect on neuromuscular transmission seemed to vary. As any neuromuscular blocking agents were not used in the present study, the interaction of remimazolam with neuromuscular blocking agents remains unclear but the direct effect of remimazolam on the TOF ratio was not determined. The physiology of neuromuscular transmission holds that the neurotransmitter acetylcholine is released from the nerve endings by nerve impulses and that it acts on the nicotinic acetylcholine receptors at the neuromuscular junction [13]. The TOF fade expressed by the TOF ratio is caused by presynaptic acetylcholine receptor blockade [14]. As no significant decrement in the TOF ratio after remimazolam administration was found in the present study, it was also suggested that remimazolam affected neither acetylcholine receptors nor neuromuscular transmission.

On the other hands, in the present study, a decrement in the first twitch response was found. The twitch response is decreased by not only inhibition of neuromuscular transmission but also depression of muscle contractions itself. Although there is no report investigating the direct depressor effects of remimazolam on muscle contractions, successful recording of motor-evoked potentials (MEPs) under general anesthesia maintained with remimazolam has been reported in previous case reports [15–18]. Particularly, Yamada et al. reported a case series showing stable MEPs with transcranial electrical stimulation of three patients in which the anesthetic agent was changed from propofol to remimazolam, as in the present study [18]. In addition, it is unlikely that the centrally acting muscle-relaxant effects of benzodiazepines including remimazolam are involved in the muscle contraction induced by peripheral nerve stimulation of TOF monitoring. Therefore, we considered that a decrement in the first twitch response found in the present study might be caused by muscle fatigue or excessive overload to the local muscle due to continuous TOF stimulation every 15 s over 60 min. However, continuous TOF stimulation every 15 s seemed to have no effect on the TOF ratio because all four responses to the TOF stimulation were decreased to the same extent.

Conversely, repeated TOF stimulation at 15-s intervals is known to result in an increment in the twitch response (the staircase phenomenon), and it takes 5–20 min to achieve a stable response [7, 19]. In the present study, although adequate stabilization periods were secured and stable TOF responses could be confirmed, a prior 50-Hz tetanic stimulation for 5 s to calibration, known to shorten the stabilization period to 2–5 min [7, 19, 20], was not applied. This may be one of the limitations in the present study, but the staircase phenomenon does not affect the TOF ratio [19]. Therefore, the repeated TOF stimulation was not considered to be related to the result about the baseline TOF ratio.

Another limitation of this study is the dose-dependency not being investigated. According to the drug manufacturer's recommendations, for usual induction of general anesthesia, remimazolam is continuously injected at 12 mg/kg/h (loading dose) until loss of consciousness. Since a case of temporarily decreased MEPs after a loading dose of remimazolam administration was reported [18], a high dose of remimazolam may affect the TOF ratio. In addition, the infusion rate of the study drugs including remifentanil during the period of collecting TOF data was not fixed in the present study, suggesting that the plasma and effect–site concentrations of remimazolam were changing. Increased blood pressure or increased pulse rate caused by pain stimulation due to surgery and some demographics of patients (e.g., age, muscle mass, and body fat percentage) may also change the plasma and effect–site concentrations of remimazolam. Further investigation is needed but it was demonstrated that remimazolam within the usual maintenance dose based on BIS value did not affect the baseline TOF ratio.

## 5. Conclusion

No significant changes in the TOF ratio between remimazolam anesthesia maintenance and propofol anesthesia maintenance were demonstrated, suggesting that switching anesthesia from propofol to remimazolam does not affect the baseline TOF ratio.

## Figures and Tables

**Table 1:** Demographic data of the patients (*n* = 12).

Age (yr)	66.6 ± 23.6
Sex (male/female)	3/9
Height (cm)	155.2 ± 7.6
Weight (kg)	57.0 ± 7.2
BMI (kg/m^2^)	23.7 ± 3.0
Electrical stimulation current for TOF monitoring (mA)	40.9 ± 11.5

*Note:* Data were expressed as mean ± standard deviation or number of patients.

Abbreviations: BMI, body mass index; TOF, train-of-four.

## Data Availability

The data that support the findings of this study are available from the corresponding author upon reasonable request.
